# An Unusual and Rare Location of Intra-articular Rheumatoid Nodule in the Elbow Joint - A Case Report

**DOI:** 10.7759/cureus.36747

**Published:** 2023-03-27

**Authors:** Sivaganesh Porko, Chandan Chowdhuri, Ashwinkumar K Barsagade, Shanmuga Priya, Mohammed Mustafa

**Affiliations:** 1 Department of Pathology, Andaman & Nicobar Islands Institute of Medical Sciences (ANIIMS), Port Blair, IND; 2 Department of General Surgery, Andaman & Nicobar Islands Institute of Medical Sciences (ANIIMS), Port Blair, IND

**Keywords:** palisading histiocytes, elbow joint, intra-articular, rheumatoid arthritis, rheumatoid nodule

## Abstract

Rheumatoid nodules are the most common extra-articular manifestation of rheumatoid arthritis. Intra-articular rheumatoid nodules are very rare and usually associated with clinical symptoms. Case reports of intra-articular rheumatoid nodules in the knee joint, wrist joint, ankle joint, and sacrococcygeal joint are reported. However, an intra-articular rheumatoid nodule in the elbow joint has not been reported in the literature. Herein, we report a unique and rare case of a symptomatic intra-articular rheumatoid nodule in the elbow joint of a 49-year-old female with a 15-year history of rheumatoid arthritis. The symptoms resolved after surgical excision of the nodule.

## Introduction

Rheumatoid arthritis (RA) is a systemic autoimmune inflammatory disease occurring in 0.5-1% of the population [1,2]. Women are more commonly affected than men [1]. This condition involves peripheral synovial joints of hands, feet, and wrists, which ultimately leads to articular destruction and disability [1]. More than 35% of RA patients present with extra-articular manifestations [3]. The most common extra-articular manifestations are rheumatoid nodules, usually present as solitary or multiple subcutaneous nodules, ranging from 0.2 to 5 cm, with the characteristic pathological finding of areas of fibrinoid necrosis surrounded by palisading histiocytes [4]. Intra-articular rheumatoid nodules are rare. Case reports of intra-articular rheumatoid nodules in the knee joint, wrist joint, ankle joint, and sacrococcygeal joint are reported in the literature [5-8].

Herein, we present a unique and rare case of an intra-articular rheumatoid nodule of the elbow joint.

## Case presentation

A 49-year-old female from South Andaman Island presented with left elbow pain, swelling, and restricted joint movements of six months duration. The pain started six months back, followed by restriction of movement and foreign body sensation in the left elbow. Fifteen years ago, she had presented with multiple joint pain and swelling of the wrist, elbow, and knee joints, and was diagnosed with seropositive rheumatoid arthritis with positive rheumatoid factor (RF) and anti-cyclic citrullinated peptide (anti-CCP) antibodies and was started on daily low-dose prednisone and weekly methotrexate. No details regarding radiological investigations were available at the time of diagnosis. She was found to have hypothyroidism and diabetes mellitus 10 years ago and was on regular medications. 

On clinical examination, the left elbow joint had restricted mobility, mild tenderness, and swelling. The other major and minor joints were normal. No joint deformity was noted. The thyroid gland appeared normal.

Laboratory investigations revealed positive rheumatoid factor (RF), mildly increased erythrocyte sedimentation rate (ESR - 35 mm/hour) and raised C-reactive protein (CRP - 12 mg/dl), normal thyroid profile (T4 - 9.16 microgram/dl, TSH - 3.15 microIU/ml). Antinuclear antibodies (ANA) and anti-cyclic citrullinated peptide (anti-CCP) antibodies were negative. Serum uric acid was mildly increased (6.9 mg/dl). Complete blood count showed severe anemia (hemoglobin - 6 g/dl), and other counts were within normal limits. Peripheral blood smear showed microcytic hypochromic anemia suggestive of Iron deficiency anemia. Fasting and postprandial glucose were within normal limits.

**Figure 1:**
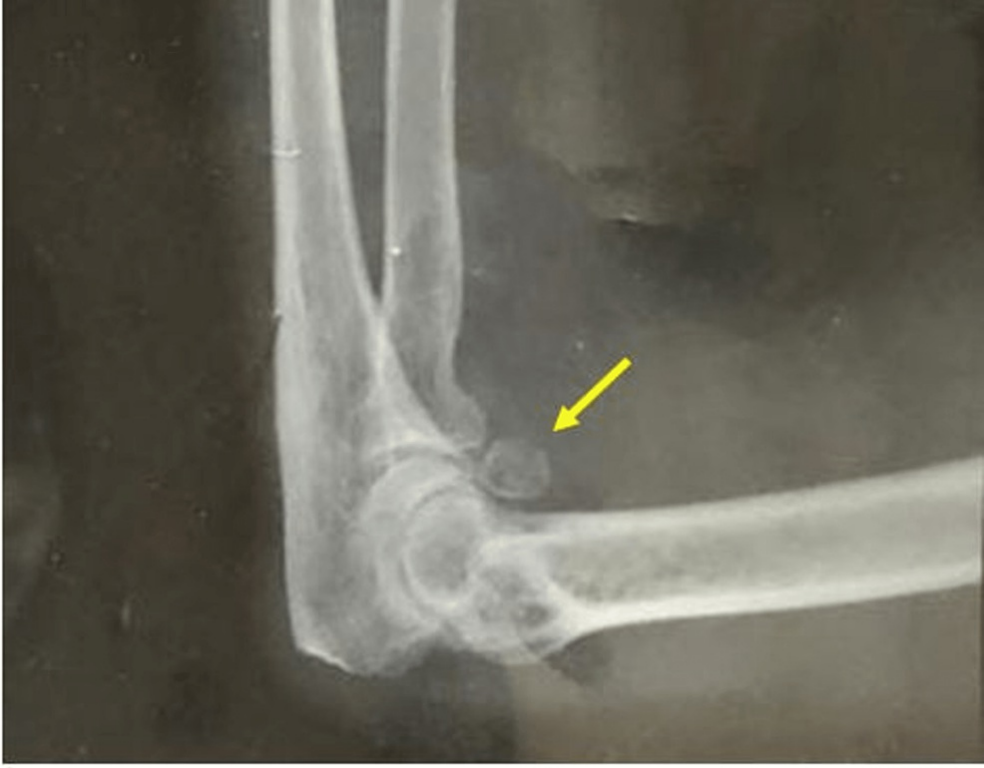
X-ray left elbow joint lateral view shows osteoporotic changes in the proximal radius, ulna, and distal humerus with narrowing of joint space and an intra-articular loose body (arrow)

X-ray lateral view of the left elbow joint showed osteoporotic changes in the proximal radius, ulna & distal humerus with narrowing of joint space and an intra-articular mass lesion/loose body (Figure 1).

A provisional clinical diagnosis of teno-synovial giant cell tumor or fibroma of tendon sheath was made, and surgery was planned to excise the mass. Intraoperatively, a hard nodule involving the elbow joint adherent to the joint capsule was excised and sent for histopathological examination. Gross examination showed three grey-white fragments of soft tissue largest measuring 1.5 x 1.5 x 1 cm.

**Figure 2:**
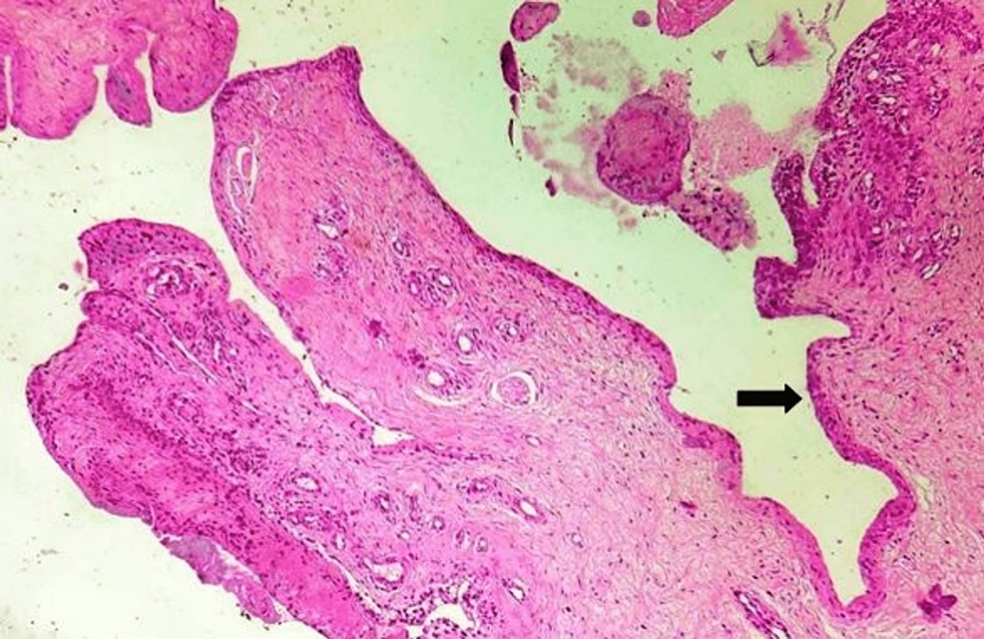
Synovium composed of fibro-collagenous tissue partially lined by hyperplastic synovial cells (arrow) and partly covered by fibrinous material (H&E stain, x40)

**Figure 3:**
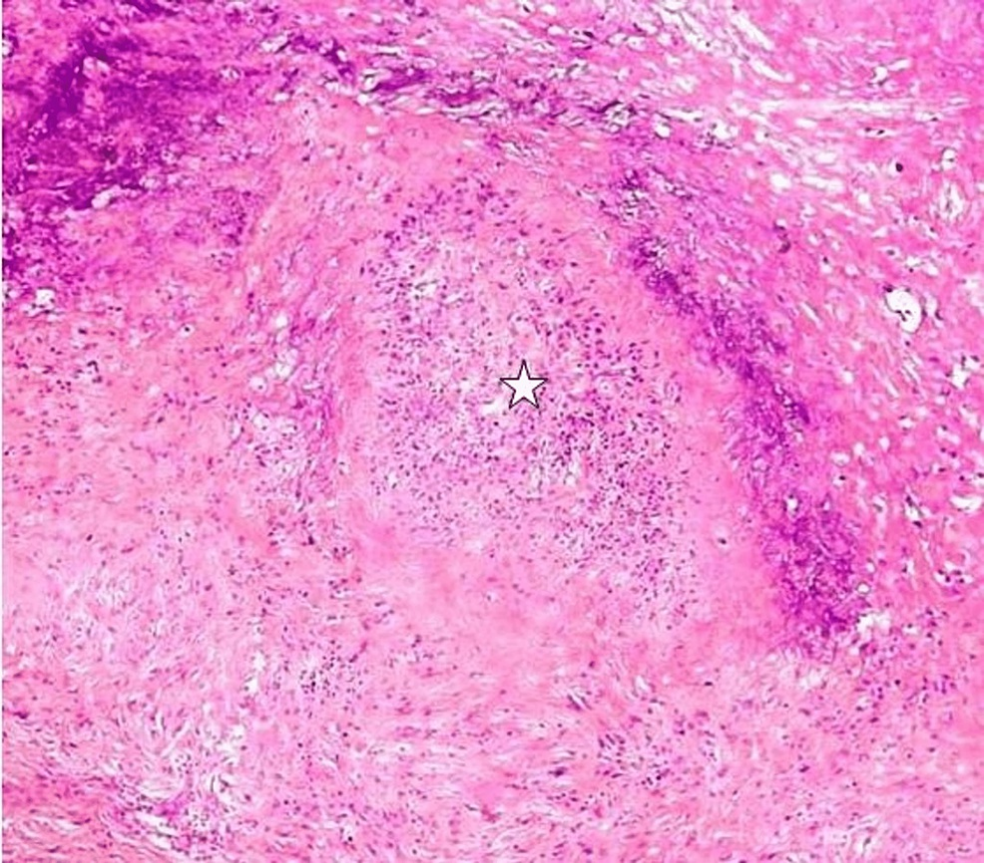
Chronic inflammatory infiltrate (star) composed of histiocytes, lymphocytes, and occasional plasma cells surrounded by amorphous fibrinous material with few palisading histiocytes and sclerosis (H&E stain, x40)

**Figure 4:**
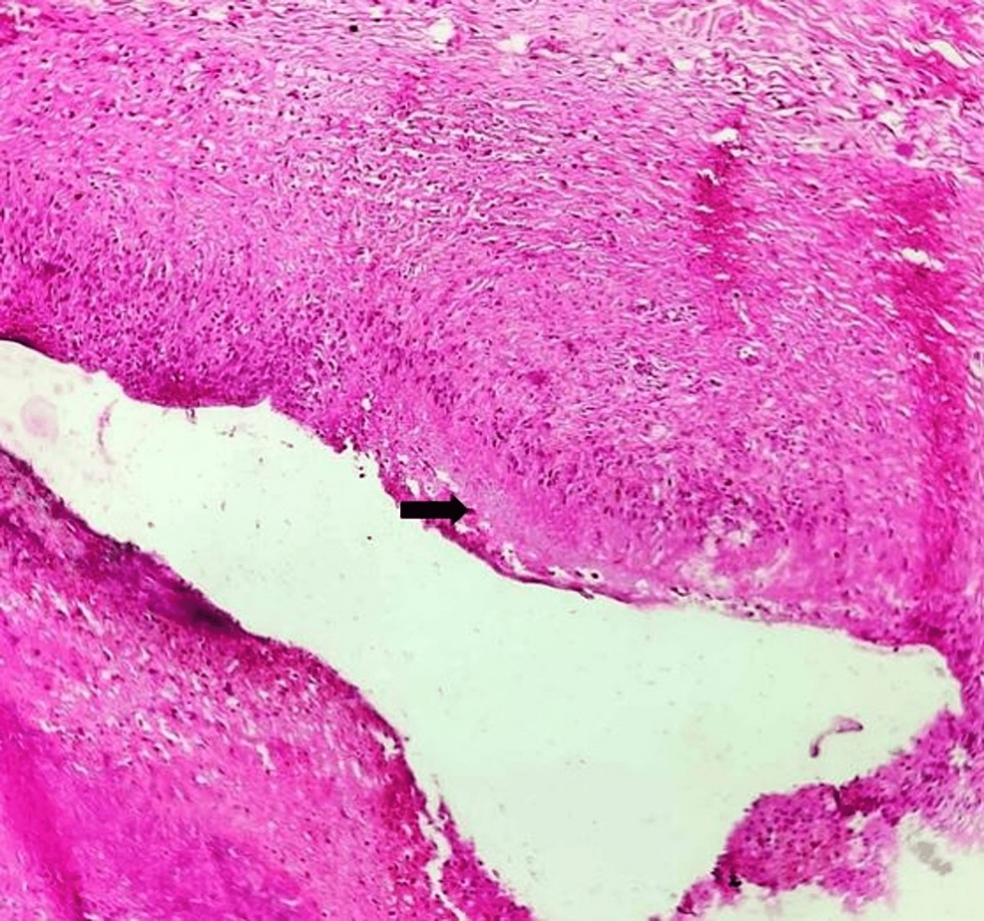
Amorphous fibrinous material (arrow) surrounded by palisading histiocytes (H&E stain, x100)

**Figure 5:**
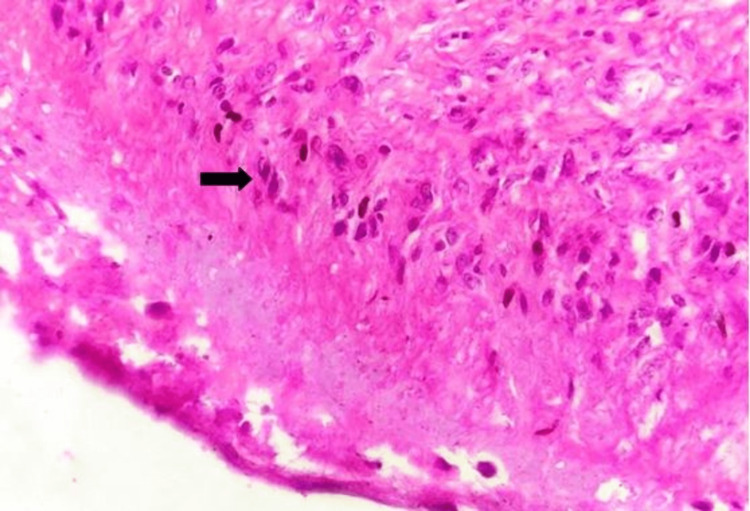
Amorphous fibrinous material surrounded by palisading histiocytes (arrow) (H&E stain, x400)

Histopathological examination showed fibro-collagenous tissue partially lined by hyperplastic synovial cells and partly covered by fibrinous material (Figure 2) with moderate chronic inflammatory infiltrate composed of lymphocytes, histiocytes, and occasional plasma cells along with few congested blood vessels (Figure 3). Palisading histiocytic aggregates around amorphous fibrinous material were noted at multiple foci (Figures 4, 5). Areas of myxoid change and sclerosis were also seen. Histopathological findings were consistent with a rheumatoid nodule.

The postoperative period was uneventful, and she was started on low-dose prednisone and allopurinol for two weeks. On regular follow-up after six months, the patient's symptoms improved with the restoration of joint mobility and reduction in joint pain with no signs of infection or recurrence.

## Discussion

Rheumatoid arthritis (RA) is a chronic autoimmune inflammatory disorder characterized by articular involvement with the presence of rheumatoid factor (RF) and rheumatoid nodules (RN). Extra-articular involvement is not uncommon in RA. Rheumatoid nodule (RN) is one of the common extra-articular manifestations of rheumatoid arthritis [9]. RNs are usually asymptomatic and are often associated with tissue trauma, elevated rheumatoid factor, anti-CCP antibodies, smoking, and methotrexate treatment [9,10]. These nodules occur in almost 20 to 35% of patients with rheumatoid arthritis and are most commonly present as subcutaneous nodules over pressure points at the elbows, the buttocks, and the scalp [10,11]. However, intra-articular rheumatoid nodules are exceptionally rare. Rare cases of intra-articular rheumatoid nodules in the knee joint, wrist joint, ankle joint, and sacrococcygeal joint are reported [5-8].

The exact pathogenesis of the development of rheumatoid nodules is unclear [11]. Multiple theories are postulated regarding the pathogenesis of the development of rheumatoid nodules. The most accepted hypothesis regarding the pathogenesis of rheumatoid nodule formation is repetitive trauma to the small vessels that may result in the accumulation of rheumatoid factors and other immune complexes, resulting in the activation of inflammation and fibrin deposition [12]. As the enzymatic and cyclic degradation of tissue is a gradual slow process, necrosis develops with palisading histiocytes surrounding the necrotic area forming a rheumatoid nodule [12].

Histopathology of rheumatoid nodules consists of central necrobiotic granuloma surrounded by a palisade of histiocytes, fibroblasts, and lymphocytes. The area of central necrosis contains a large population of HLA-DR+ staining cells, which releases interleukin (IL)-1, an important cytokine involved in the pathogenesis of RA [13]. Histopathological findings of the synovium in RA may show hyperplasia of the synovial lining cells, neo-angiogenesis, infiltration of immune cells of both the innate and adaptive immune system and fibrin deposition on the synovial surface [12,13].

Various studies showed that an interruption to methotrexate medication leads to a decrease in the size of the rheumatoid nodules [14,15]. The treatment of subcutaneous rheumatoid nodules is conservative management, and drainage is not recommended due to the risk of recurrence and infection [16]. Management of intra-articular rheumatoid nodules may be conservative, but surgical excision is indicated if the patients have disabling symptoms or infection [17]. 

Patients with intra-articular RNs are usually symptomatic with joint pain and restricted mobility, similar to this case, and excisions were done in most of these cases. One of seven cases of intra-articular RNs in the knee joint and one of two cases in the sacrococcygeal joint showed recurrence, and no recurrences were reported in the ankle and wrist joints [5-8]. On regular follow-up after six months, the patient's symptoms improved with the restoration of joint mobility and reduction in joint pain with no signs of infection or recurrence.

After an extensive literature search, an intra-articular rheumatoid nodule in the elbow joint was not found to be reported. This case represented an unusual and rare location of rheumatoid nodule occurring intra-articularly in the elbow joint of a patient with RA, and to the best of our knowledge, this may be the first case of symptomatic intra-articular rheumatoid nodule in the elbow joint reported in the literature. 

Since the rheumatoid nodule in this case presented as an intra-articular nodule/mass, other intra-articular mass lesions such as teno-synovial giant cell tumor, fibroma of the tendon sheath, and synovial chondromatosis should be excluded. Histopathological examination in such cases is essential for a definite diagnosis. Early diagnosis and timely intervention are essential to reduce complications.

## Conclusions

Rheumatoid nodules usually present as subcutaneous nodules over pressure points. Intra-articular rheumatoid nodules are exceptionally rare. This is an interesting and uniquely rare case of a symptomatic intra-articular rheumatoid nodule in the elbow joint. Histopathological examination is essential for the correct diagnosis of rheumatoid nodule and to rule out other intra-articular lesions such as teno-synovial giant cell tumor, fibroma of the tendon sheath, and synovial chondromatosis. This may be the first case of an intra-articular rheumatoid nodule in the elbow joint reported in the literature.
